# Astrocyte ethanol exposure reveals persistent and defined calcium response subtypes and associated gene signatures

**DOI:** 10.1016/j.jbc.2022.102147

**Published:** 2022-06-16

**Authors:** Hyun-Bum Kim, Youtao Lu, Seonkyung C. Oh, Jacqueline Morris, Kevin Miyashiro, Junhyong Kim, James Eberwine, Jai-Yoon Sul

**Affiliations:** 1 Department of Systems Pharmacology and Translational Therapeutics, Perelman School of Medicine, University of Pennsylvania, Philadelphia, Pennsylvania, USA; 2 Department of Biology, School of Arts and Sciences, University of Pennsylvania, Philadelphia, Pennsylvania, USA; 3 PENN Program in Single Cell Biology, University of Pennsylvania, Philadelphia, Pennsylvania, USA

**Keywords:** astrocyte, ethanol, calcium signal, single-cell transcriptomics, gene signature, phenotype, aRNA, antisense RNA, BBB, blood-brain barrier, DEG, differentially expressed gene, EtOH, ethanol, GO, gene ontology, GSEA, gene set enrichment analysis, NR, nonresponsive, PCA, principal component analysis

## Abstract

Astrocytes play a critical role in brain function, but their contribution during ethanol (EtOH) consumption remains largely understudied. In light of recent findings on the heterogeneity of astrocyte physiology and gene expression, an approach with the ability to identify subtypes and capture this heterogeneity is necessary. Here, we combined measurements of calcium signaling and gene expression to define EtOH-induced astrocyte subtypes. In the absence of a demonstrated EtOH receptor, EtOH is believed to have effects on the function of many receptors and downstream biological cascades that underlie calcium responsiveness. This mechanism of EtOH-induced calcium signaling is unknown and this study provides the first step in understanding the characteristics of cells displaying these observed responses. To characterize underlying astrocyte subtypes, we assessed the correlation between calcium signaling and astrocyte gene expression signature in response to EtOH. We found that various EtOH doses increased intracellular calcium levels in a subset of astrocytes, distinguishing three cellular response types and one nonresponsive subtype as categorized by response waveform properties. Furthermore, single-cell RNA-seq analysis of astrocytes from the different response types identified type-enriched discriminatory gene expression signatures. Combining single-cell calcium responses and gene expression analysis identified specific astrocyte subgroups among astrocyte populations defined by their response to EtOH. This result provides a basis for identifying the relationship between astrocyte susceptibility to EtOH and corresponding measurable markers of calcium signaling and gene expression, which will be useful to investigate potential subgroup-specific influences of astrocytes on the physiology and pathology of EtOH exposure in the brain.

Ethanol (EtOH) is a commonly abused psychoactive substance, and its abuse is one of the leading causes of preventable deaths in the world (1, 2). Significant disease burden arises from pathological EtOH consumption by adults (Alcohol Use Disorder or AUD) as well as from prenatal exposure that can lead to fetal alcohol syndrome. In rodents, *in utero* or neonatal exposure to even a single dose of EtOH results in significant changes to both the overall epigenetic state of DNA and in gene expression programs in populations of brain cells that persist into later developmental stages (3, 4, 5, 6, 7). These epigenetic and transcriptional changes impact a number of cellular functions, including calcium signaling, protein and lipid processing in the endoplasmic reticulum, endosomal or lysosomal sorting and endocytosis, autophagy, and stress responses (8, 9, 10, 11, 12). The behavioral effects of EtOH self-administration in rodents have been investigated as well as the effects of EtOH on physiology, epigenetics, and gene expression from populations of brain cells, but with an emphasis on neuronal function (9).

In the brain, the astrocyte is known as the most abundant glial cell types with a functional role of metabolic and structural support for brain activity. Astrocytes form the blood-brain barrier (BBB) associated with endothelial cells and structurally serve as a link between neurons, supporting brain function and metabolism by exchanging metabolites into and nutrients from the bloodstream (13, 14). Therefore, astrocytes are readily exposed to BBB-permeable circulating compounds, such as EtOH, in the bloodstream. The chemical characteristic of EtOH as a small lipophilic molecule can easily distribute into and through lipid compartments. It passes the protective BBB into the brain and affects the function of diverse cell types (15). However, the reactivity of astrocytes to EtOH is not clear yet. The evidence of the reciprocal signaling between neurons and astrocytes has dramatically changed the classical view that astrocytes play a passive role in brain function (16, 17, 18). The presence of various neurotransmitter receptors and the capability of releasing neurochemicals from astrocytes highlights a significant active contribution to neuronal function (19). As a consequence of lacking electrical excitability, the cellular activity of astrocytes is widely evaluated by calcium signals. In general, the role of calcium signaling has cognate changes interlinked with gene regulation, biochemistry, and cell fate (20). The upregulation of intracellular calcium levels in astrocytes is known to trigger chemical release, which can facilitate intercellular signaling to neighboring cells, including adjacent astrocytes and neurons, and further influence vasoactivity (21). Recently, subtypes of astrocyte have been suggested based on physiology and transcriptomics, but the functional role of previously identified subtypes is not clear yet (18).

Expanding the knowledge of the impact of EtOH on intracellular signaling in astrocytes is likely to provide insight into the underlying mechanisms that contribute to these systemic effects of EtOH on brain function (22). However, measuring the direct impact of EtOH exposure on single cells *in vivo* is difficult due to the indirect effects through numerous complex connections and secondary effects communicated by other cells in the network, making it difficult to isolate direct effects. For example, a single astrocyte is estimated to contact thousands of neurons by ensheathing synapses *in vivo* (23). As such, *in vitro* primary cell culture provides a controlled environment that allows characterization of the direct effect of EtOH exposure on astrocytes. Under these conditions, any observed effects of EtOH exposure on this single astrocyte are due to the direct impact of EtOH. It was recently demonstrated in an all *in vitro* model that EtOH facilitates cell-to-cell communication and enhances calcium signals in both neurons and astrocytes (24). Here, we report stable and persistent calcium responses to EtOH along with correlated gene expression signatures that highlight baseline gene expression differences responsible for mitochondrial function, calcium homeostasis, and inflammation. Using classification of astrocyte calcium responses to EtOH as a selection strategy for single-cell gene expression analysis highlights unique gene signatures for each subset of astrocytes, providing previously obscured insight into heterogeneous cellular properties underlying the various effects of acute EtOH exposure.

## Results

### Acute EtOH exposure reveals distinct classes of calcium responses in a subset of astrocytes

**Figure 1:**
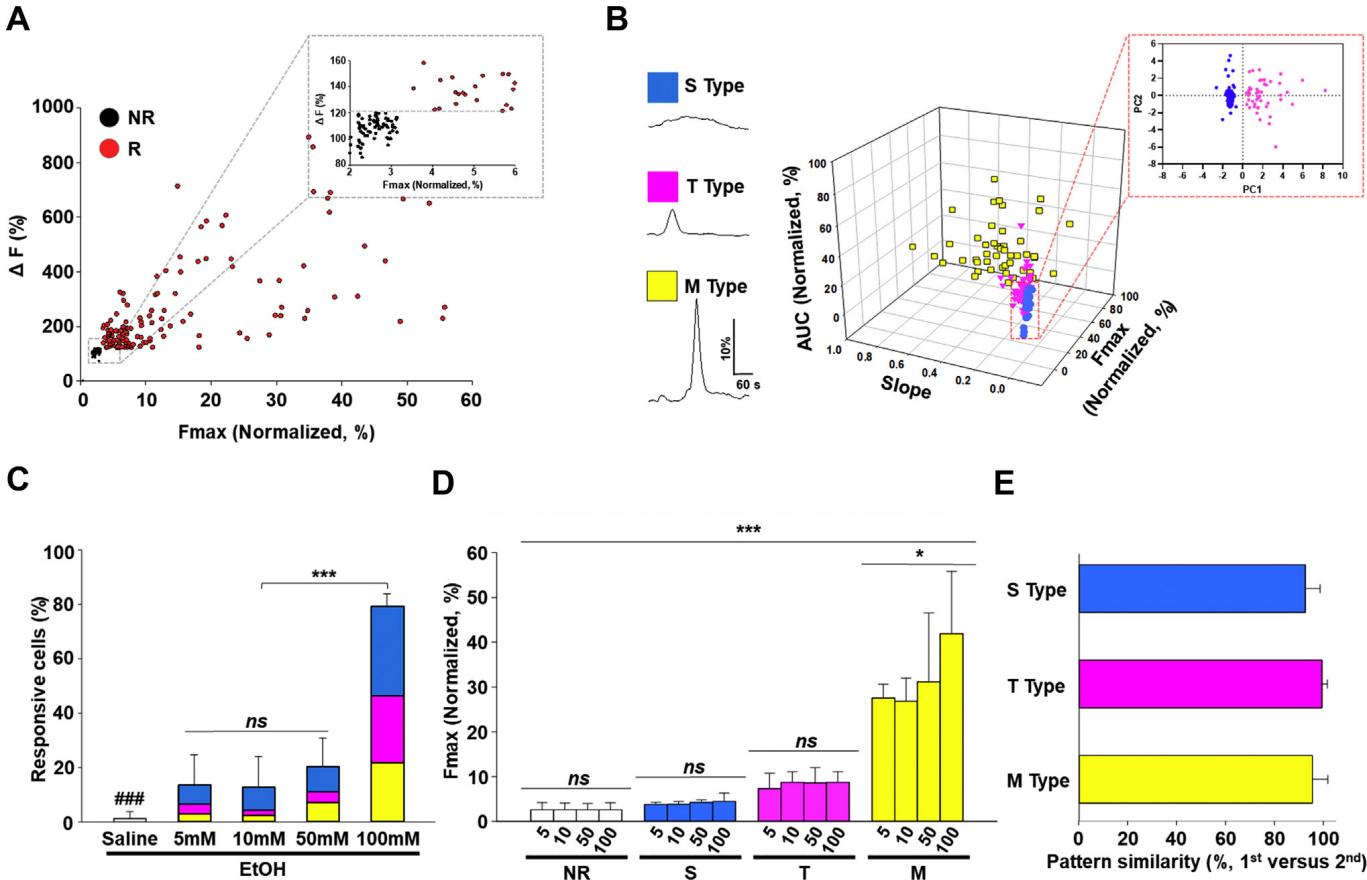
**Characteristics of different types of astrocyte calcium responses to EtOH.***A*, representative astrocyte calcium responses in enriched primary astrocyte culture classified according to the calcium signal caused by a 3 min EtOH treatment (100 mM). The astrocyte responsiveness threshold between response (R) and nonresponse (NR) is defined as an increase of 20% signal over baseline fluorescence (F_o_). *B*, representative calcium signal traces over time (ΔF) are shown on the x-axis with the percent change from baseline indicated on the y-axis. Type S: slow increase and slow decay, type T: transient increase, type M: mixed type. A dot plot of these astrocyte response types separated by the maximum (F_max_), area under the curve (AUC), and initial slope (slope %) of the calcium response is shown to the right of representative traces. S-types are defined by slope steepness < 0.1 and F_max_ < 20% (*blue circles*), T-types by slope steepness > 0.1 and F_max_ < 20% (*magenta triangles*), M-types by slope steepness > 0.1 and F_max_ >20% with a variable waveform (*yellow squares*, n = 47 cells, 3 animals). The enlarged area enclosed by a *dotted red square* emphasizes separation of types S (*blue*, n = 94 cells, 3 animals) and T (*magenta*, n = 64 cells, 3 animals) by principal component analysis using defined parameters. *C*, proportion of astrocytes responding with increased intracellular calcium to different doses of EtOH. Proportion of each astrocyte response type defined in (B) observed at different EtOH doses (color-coded) (n = 62 cells (0 mM, saline, 3 animals), n = 88 cells (5 mM, 3 animals), n = 291 cells (10 mM, 4 animals), n = 247 cells (50 mM, 4 animals), n = 125 cells (100 mM, 3 animals)). *D*, the average maximum of calcium responses (F_max_) of each response type (S, T, and M) and fluorescence signal in nonresponsive astrocytes (NR) shows stability of calcium signals in classified types at different EtOH doses (5,10, 50, and 100 mM, 14 Animals). *E*, stability of astrocyte response types is demonstrated by retention of the calcium signaling patterns following a second dose of EtOH. Indicated is the percentage of astrocytes responding to the second EtOH treatment with the same waveform type as the response to the first. Bars are color-coded as in other panels in figure. Data are presented as mean± SD. *ns*, not significant. ∗*p* < 0.05. ∗∗∗*p* < 0.001. ^###^*p* < 0.001. Additional statistical details are provided in Table S6. EtOH, ethanol.

In general, the level of blood alcohol concentration classified as low dose is 2 to 20 mM (social drinking level: 5–10 mM; driving under the influence level: 15 to 20 mM), medium dose is 20 to 50 mM (binge drinking level), and high dose is 50 to 100 mM. The high dose is not experienced by normal individuals but is frequently reached by chronic alcoholics. Even the extreme 100 to 200 mM range would be lethal to normal individuals but not to alcoholics (25). Calcium signaling is ubiquitous in cellular functions. Thus, it is widely used to investigate cellular status from physiology to pathology in many cell types and is especially useful in electrically silent cells like astrocytes. Cellular responsiveness to EtOH exposure in a single astrocyte was measured by monitoring the intracellular calcium signals in primary cultures enriched for astrocyte growth with the fluorescent calcium indicator, Fluo-4 (26, 27). An astrocyte calcium response was defined as a 20% fluorescence signal increase above the baseline fluorescence signal (Fig. 1*A*). The 20% threshold was projected by the Wilcoxon signed-rank test based on fluorescence signal fluctuations from all experimental samples, including controls. Astrocyte calcium response types were classified following the acute application of EtOH (5–100 mM, 3 min) based on the following reproducibly observed features of the EtOH-induced calcium signal: steepness (slope), amplitude (F_max_), and peak frequency in waveforms (Fig. 1*B*, traces). Three distinct calcium signal patterns were identified and designated as S-type (sustained signal; slow signal increase and decay), T-type (transient signal; sharp signal increase and decay), or M-type (mixed signals; complex signal patterns that include the characteristic of S- and T-types). M-type responses consisted of a variable mixture of S- and T-type characteristics but with significantly higher peak amplitude (Fig. 1*B*, traces). The validation of signal classification parameters of response types was also confirmed using principal component analysis (PCA) (Fig. 1*B*, trace, insert and Fig. S1). These distinct signal patterns were observed across a range of EtOH concentrations representative of those achieved with casual to sublethal consumption (5–100 mM). Although maximal responsiveness was achieved by the 100 mM dose (Fig. 1*C*), response amplitudes for each response type were consistent across EtOH doses with only M-type showing a trend of increasing amplitudes with increasing EtOH dose (Fig. 1*D*). For each EtOH dose, the induced specific calcium signal returned to baseline following completion of the EtOH treatment, emphasizing that these are cell-specific responses and ruling out the possibility that these are nonspecific or catastrophic intracellular calcium elevation caused by EtOH exposure (Fig. 1*A*). S- and T-type responses were observed for each dose of EtOH (Fig. 1*D*) and persisted with repeated EtOH exposure (Fig. 1*E*, 96.1% ± 2.6 of cells maintained the same response pattern across sequential EtOH applications with 6 min washing intervals) indicating that these are stable calcium signal phenotypes. While M-type responses were also persistent and stable in response to repeated EtOH exposure, the trend of increasing maximum amplitude with increasing EtOH dose (Fig. 1*D*, yellow plots) suggests a malleability of M-types that is distinct from S- or T-type responses. Also, given that the astrocytes were in low-density dispersed culture, these subtype distinctions are cell specific and not the result of cellular connectivity.

### Single-cell RNA-seq correlates gene signatures with astrocyte calcium signal phenotypes

Based on the distinct and classifiable calcium signal patterns of the S-, T-, and M-type calcium responses to EtOH exposure, we investigated whether or not differences in gene expression between these subtypes could be associated with these distinct calcium responses. To do so, single astrocytes exhibiting an identified calcium signal type were immediately isolated using a micropipette (less than 2 minutes after calcium signal type identification) at the completion of the fluorescence imaging and online signal analysis. The single-cell samples were snap frozen and subjected to subsequent RNA amplification for sequencing and bioinformatics analysis. Briefly, the single astrocyte RNA was amplified by the quasi-linear antisense RNA amplification method and libraries constructed using the Illumina TruSeq mRNA stranded kit per the manufacturer’s instructions and libraries sequenced at high depth on the NextSeq 500. For analysis, reads were demultiplexed based on the library-specific barcode, trimmed to remove low-quality bases and barcode sequence prior to alignment to the reference genome with STAR and gene quantification with VERSE (28). The raw read counts (Table S1) were normalized, and differential expression was performed with the DESeq2 R package (29). The 100 mM EtOH dose was selected for experiments since it elicits the highest response probability for S-, T-, and M-types (Fig. 1*C*). Given these experimental conditions of 100 mM EtOH exposure, differential expression analysis of single astrocytes collected at 3 or 60 min after acute EtOH exposure (3 min) was first compared against canonical apoptotic gene sets derived from public databases (www.gsea-msigdb.org/gsea/msigdb/cards/HALLMARK_APOPTOSIS, http://amigo.geneontology.org/amigo/term/GO:0097194, and www.gsea-msigdb.org/gsea/msigdb/cards/KEGG_APOPTOSIS) to ascertain if these stimulus paradigms initiated any significant evidence for activation of the apoptotic process. Out of 234 apoptotic genes derived from these curated gene sets, only four were differentially expressed between samples collected at 3 *versus* 60 min (*Satb1*, *Tgfb2*, *Timp1*, *Nfkb1*) (http://www.gsea-msigdb.org/gsea/msigdb/cards/KEGG_APOPTOSIS and http://amigo.geneontology.org/amigo/term/GO:0097194) (30). These results suggest that the stimulation protocol used in this study as 3 min stimulation by 100 mM EtOH is not acutely toxic and did not induce an apoptotic process in astrocytes (Table S2).

**Figure 2:**
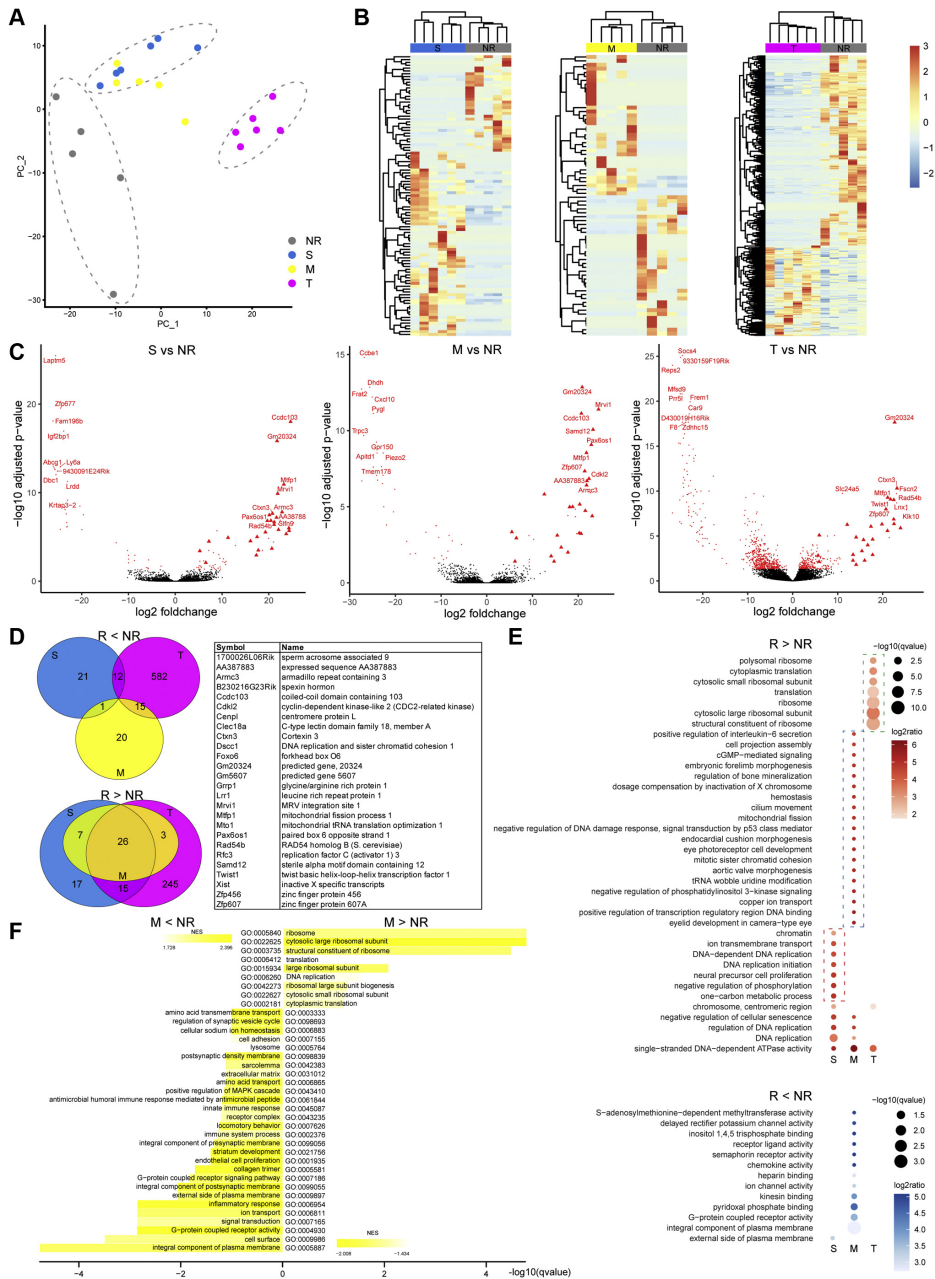
**Differential expression of EtOH responding (R) subtypes S, M, and T in contrast to the baseline (NR).***A*, PCA visualization differentially expressed genes derived from contrasting each of the R- to the NR-type. Samples are colored by the subtype and the NR-, T-, and the mixture of M- and S-types are highlighted by the *dashed-line ovals*. *B*, heatmaps showing upregulated and downregulated gene expression for subtype comparisons: S *versus* NR (*left*), M *versus* NR (*middle*), T *versus* NR (*right*). Each column in the heatmap represent the gene expression levels for each single cells analyzed in each category indicated in A. The color represents z-scores for each gene. *C*, volcano plots showing the significantly differential genes (*red dots*) in subtype comparisons: S *versus* NR (*left*), M *versus* NR (*middle*), T *versus* NR (*right*), top 10 differential genes (in either direction) shown in *red* font, the 26 intersection genes shown as *red triangles*. *D*, venn diagrams show the shared and unique genes differentiating R from NR. *Top left*, genes higher in NR; *bottom left*, genes higher in R; *right*, gene annotation for the 26 R > NR intersection genes. *E*, hypergeometric test–based Gene Ontology (GO) enrichment for the differential genes in subtype comparisons. *Top*, genes higher in R; *bottom*, genes higher in NR. *F*, GSEA for the differential genes comparing M *versus* NR. *Top*, genes higher in M; *bottom*, genes higher in NR. EtOH, ethanol; GSEA, gene set enrichment analysis; NR, nonresponsive; PCA, principal component analysis.

To investigate gene expression signatures for each calcium response type in astrocyte subgroups, whole transcripts of each type were compared to the other types, and significantly differentially expressed genes (DEGs) were identified by DESeq (29). First, each of the three responsive subtypes were compared against the nonresponsive astrocytes (NR) subtype. The union of the differential genes were used for PCA, which illustrates the different distribution of S-, M-, T-, and NR astrocyte clusters in the 2D plot (Fig. 2*A*). When compared to NR astrocytes, heatmap depictions of the differences in gene expression typify the underlying transcriptomic signatures of S-, M-, or T-type clusters (Fig. 2*B*). With further quantitative analysis, the astrocytes in different response types showed significant upregulated and downregulated gene signatures with a large gene expression variation in T-type astrocytes (Fig. 2*C*). Interestingly, these bioinformatics analyses also identify a core set of 26 upregulated genes across response types that may be a defining transcriptomic motif/pattern for calcium responsiveness in astrocytes. In contrast, independent type-specific downregulated genes may refine type-specific calcium response (Fig. 2*D*). The functional enrichment of DEGs of each response type was investigated using gene ontology (GO) enrichment analysis. The upregulated DEGs across S-, T-, and M-types showed the enrichment of translation-related functions in T-type, broad cell division related functions (*chromatin, DNA replication, and cell proliferation*) in S-type, and broad biological function-related features in M-type astrocytes (Fig. 2*E*).

**Figure 3:**
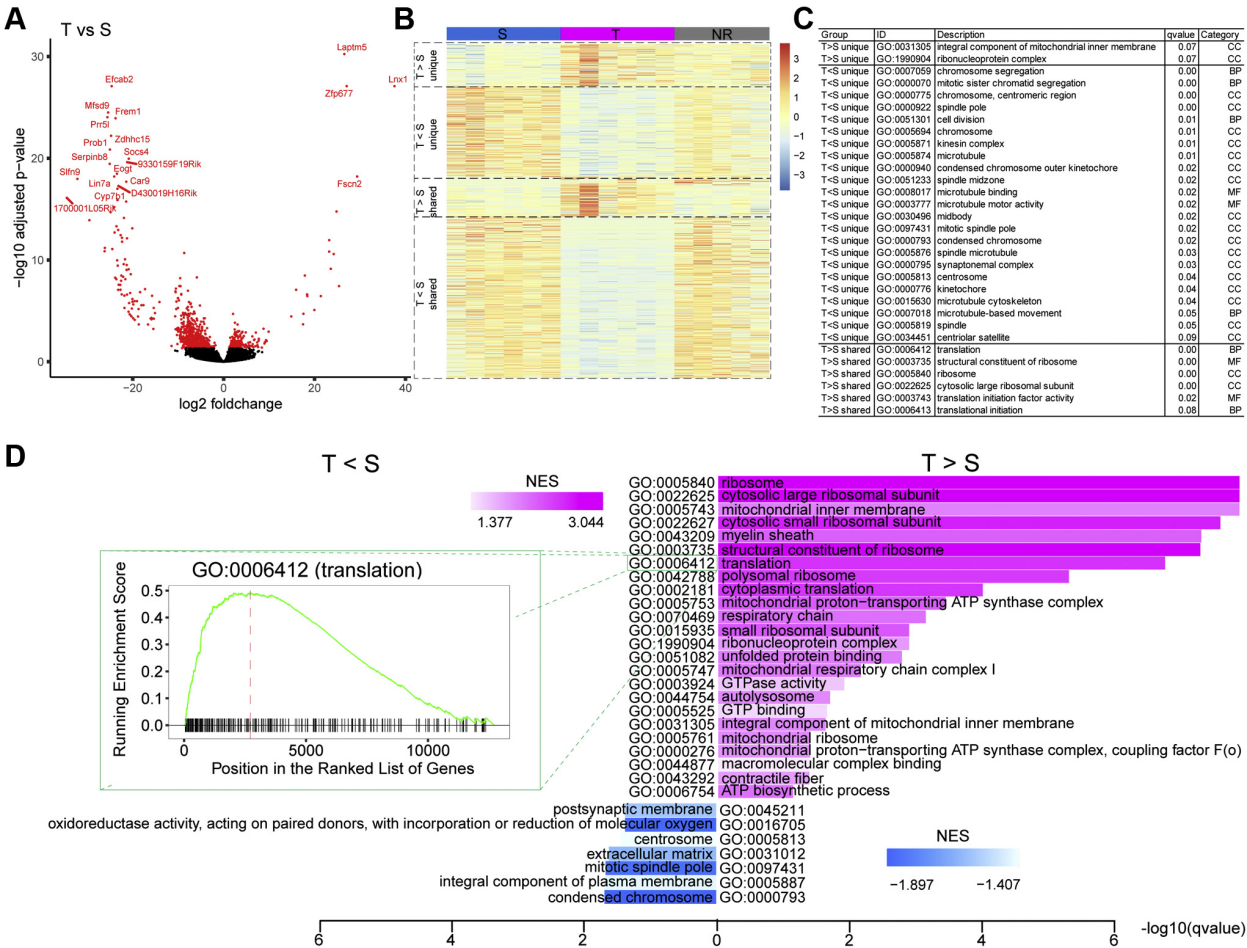
**Differential expression between the S- and T-type, general Gene Ontology pathways.***A*, volcano plot showing the significantly differential genes (*red dots*) between T and S, top genes shown in *red* font. *B*, heatmap showing the expression level (in z-scores) in S, T, and NR. Genes are grouped into four classes: (1) T > S or (2) T < S unique, the differential genes in T *versus* S but not in T *versus* NR or S *versus* NR; (3) T > S shared, the differential genes where expression in T > S and in T > NR or S < NR; (4) T < S shared, the differential genes where expression in T < S and in T < NR or S > NR. *C*, hypergeometric test–based GO enrichment for the differential genes defined in (B). GO terms were collected from three categories: biological processes (BP), molecular functions (MF), cellular components (CC). *D*, GSEA for the differential genes comparing T *versus* S. The *upper barplot* shows the significantly enriched GO terms in T > S (*magenta*), while the *lower barplot* shows the significantly enriched GO terms in T < S (*blue*). The color gradient represents the Normalized Enrichment Score (NES). The length of the bars represents the *q*-value in logarithm. The inset shows the GSEA running-sum statistic (*green curve*), the genes overlapping the target gene set (*gray whiskers*), and the leading-edge hits (*red dashed line*) for the particular pathway: GO:0006412 (translation). GO, gene ontology; GSEA, gene set enrichment analysis; NR, nonresponsive.

Compared to the stable calcium signal patterns observed in S- and T-types, which are persistent with repeated EtOH exposure across different EtOH doses, M-type signal is characterized by the inconsistency of signal patterns (mixture of other types). Of the identified DEGs, upregulated genes in M-type astrocytes completely intersected with those identified in S- and T-types (Fig. 2*D*, lower panel). Thus, the gene expression signatures of S- and T-type could reveal new genetic markers for these particular subpopulations of astrocytes. Taking advantage of the coherent calcium signals and distinct DEGs of S- and T-types compared to NR, identification of response type-specific gene expression was further investigated. Based on a pairwise comparison between types, at the minimum foldchange of two and the maximum Benjamini-Hochberg corrected *p*-value of 0.05, the DESeq2 Wald test for differential gene expression revealed 849 DEGs in T- *versus* S-type (Fig. 3*A*), 898 in T- *versus* NR-type, and 99 in S- *versus* NR-type (Table S3). To further explore the relationship between the DEGs, we compared the aggregate list of all DEGs, comprising both upregulated and downregulated genes, identified as T-specific (Fig. S2*A*) or S-specific types (Fig. S2*B*) from a pairwise comparison of the T-type *versus* S-type RNA-seq datasets. When annotated this way, T-types express 107 distinct DEGs compared to S-types, and S-types express 230 DEGs that are unique to S-types when compared to T-types. We then identified aggregate lists of DEGs, which varied widely in number, found in other pairwise comparison of RNA-seq datasets. For example, there could be as many as 597 (*i.e.*, NR-specific DEGs identified that are expressed in NR cells when compared to T-types (T<NR; Fig. S2*A*)) or as few as 5 (*i.e.*, NR-specific DEGs identified that are expressed in NR cells when compared to S-types (S<NR; Fig. S2*A*)) when normalized against the T-specific DEGs (T > S). The diagrams comparing DEGs for each of the pairwise comparison to the T-specific DEGs demonstrate the frequency that T-type–specific DEGs are shared in any of the other pairwise comparisons. We observed that, out in addition to the 107 genes from the T > S comparison, another 201 DEGs uniquely expressed in T-types were identified *via* comparisons against other pairwise lists of aggregate DEGs (*i.e.*, the sum of 82 shared genes from the comparison of T > S *versus* T>NR, 17 genes from the comparison of T > S *versus* S<NR, and 2 genes from the comparison of T > S *versus* both S<NR and T<NR). It is notable that 82 or 39% of the total of the T-specific DEGs are shared with the DEGs expressed by T-types when compared to NR types (T>NR) while only two (*Fam196b*, *Igf2pb1*) overlap with DEGs expressed by NR types compared to T-types (T<NR; Fig. S2*A*), which suggests a clear demarcation in the molecular distinctiveness of the T-types compared to the S- and NR-types. In a similar analysis of S-type–specific DEGs, we find 230 unique DEGs when comparing S-type to T-Types (T < S; Fig. S2*B*). An additional 411 S-type–specific DEGs can be identified (*i.e.*, sum of 397 genes from the comparison of T < S *versus* T<NR, 12 genes from a comparison of T < S and S>NR, and one each from the comparison T < S *versus* two other pairwise combinations). In contrast to the T-type–specific analysis, S-types (T < S) display a less discrete molecular identity. They share nearly 400 DEGs when compared to T<NR which emphasizes their dissimilarity with T-types. However, only 2% of the total DEGs, or 13, show overlap when the S-specific pairwise (T < S) is compared against S-specific DEGs identified when compared to NR types (S>NR; 12 DEGs) and one (*Reps2*) from another pairwise comparison (S<NR; Fig. S2*B*). The lack of overlap observed here suggests that S-types, though clearly expressing a different transcriptomic landscape than T-types, have a less distinctive molecular signature than T-types with both showing differing degrees of distinctiveness from NR types. The general GO enrichment analysis based on hypergeometric test reveals that T-type astrocytes are functionally enriched in translation-related genes such as *ribonucleoprotein complex* in GO category cellular component, *structural constituent of ribosome* and *translation initiation factor activity* in molecular function, and *translational initiation* in biological process (Fig. 3*C*). In comparison, S-type astrocytes are enriched in structural component-related pathways such as *chromosome segregation* and *cell division* in biological process, *centrosome*, *kinetochore*, and *microtubule* in cellular component (Fig. 3*C* and Table S4). As confirmation, we repeated the enrichment analysis using gene set enrichment analysis (GSEA) (31). Consistent with the findings mentioned above, *ribosome*, *cytosolic large ribosomal subunit,* and *mitochondrial inner membrane* are the top enriched functions in T-type, while *condensed chromosome* and *mitotic spindle pole* are among the top enriched functions in S-type (Fig. 3*D* and Table S4). In M-type, besides complete intersection of upregulated genes with S- and T-types (Fig. 2*D*, lower Venn diagram), downregulated genes in particular were also shared with NR astrocytes where gene function is related to cellular physiology such as receptors, membrane functions, and signaling pathways (Fig. 2*F* and Table S5). Taken together, the distinct gene expression and calcium signal of T-types suggest it is the most distinct phenotype among EtOH responsive astrocytes.

### S- and T-types differentially express inflammatory factors and regulators of intracellular calcium, mitochondrial function, and the epigenome

**Figure 4:**
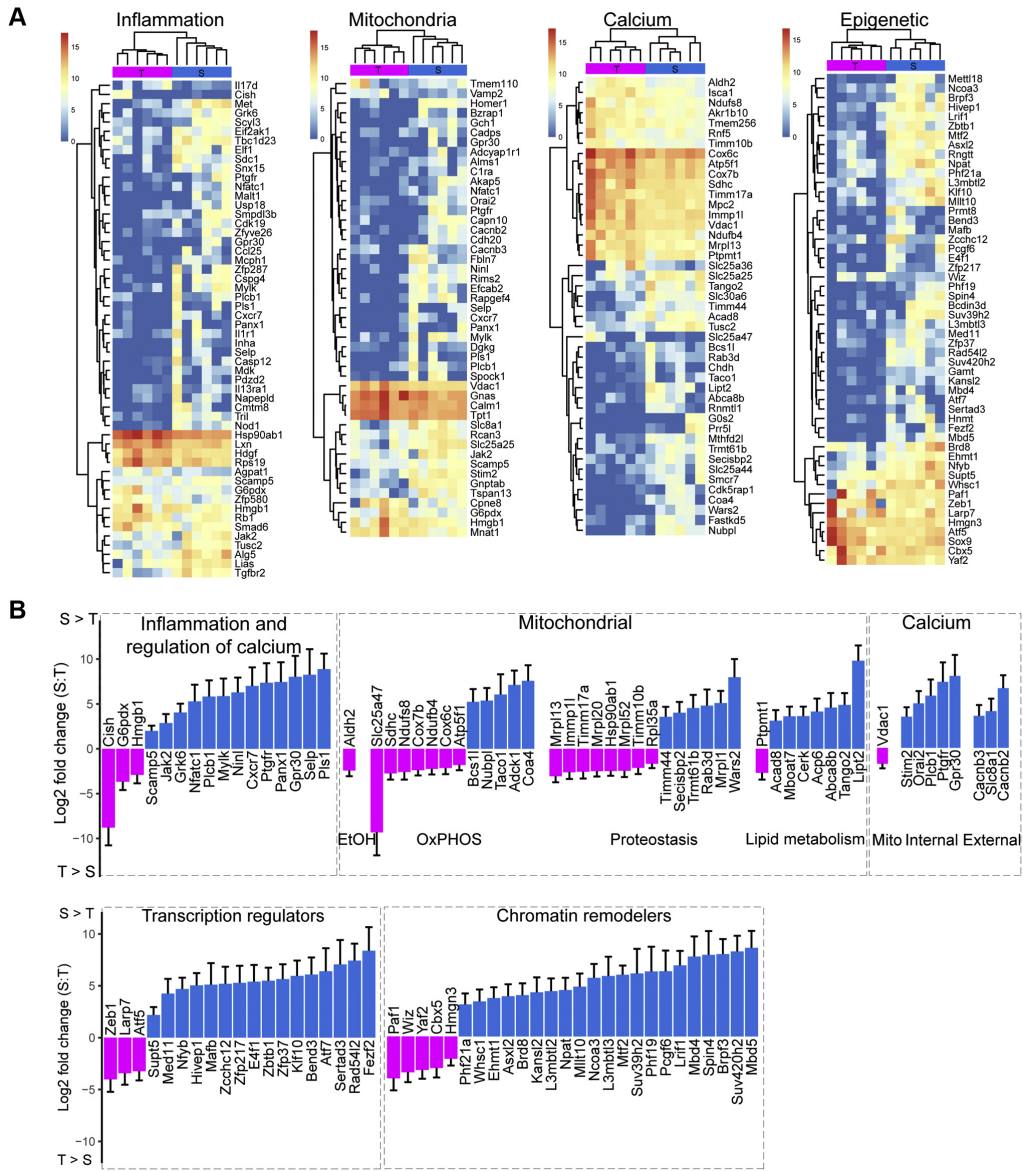
**Differential expression between the S- and T-type, selected specific pathways: inflammation, mitochondria, calcium regulation, and epigenetic factors.***A*, heatmaps showing the expression level (in z-scores) of the genes comparing S- and T-type in four selected pathways: inflammation, mitochondria, calcium and epigenetic factors. *B*, barplots showing individual differential genes comparing S- and T-type in the selected pathways from (A) plus the chromatin remodelers. Y-axis, log2 foldchange (S > T, blue; T > S, magenta).

GO enrichment analysis of genes differentially expressed across all contrasts indicates clear functional correlations across DEGs in each response type. In order to understand the higher-order molecular organization of these different response types, genes with GO term annotations in selected pathways that are functionally correlated to cellular calcium signaling were further examined to identify functionally relevant differences in the expression landscape between S- and T-type astrocytes. S-type and T-type contrasts with NR were focused on due to their distinct and uniform calcium waveform properties and corresponding distinct DEG functional classes identified by GO term analysis and GSEA. Inflammatory response: Of the 54 genes differentially expressed between S- and T-types involved in the regulation of inflammatory responses (Fig. 4*A*: inflammation, Table S5), 16 of these genes can also regulate intracellular calcium levels (*Jak2*, *Nfatc1*, *Plcb1*, *Mylk*, *Grk6*, *Cish*, *G6pdx*, *Cxcr7*, *Hmgb1*, *Ptgfr*, *Selp*, *Gpr30*, *Scamp5*, *Ninl*, *Pls1*, *Panx1*) (Fig. 4*B*: inflammation-regulation of calcium). All but three of these genes (*Cish*, *G6pdx*, *Hmgb1*) are enriched in S-type astrocytes compared to T-type astrocytes, suggesting pathways that regulate inflammatory responses active at baseline might contribute to shaping the calcium waveform in response to EtOH. *G6pdx* is believed to be involved in responses to EtOH, potentially due to its role in regulating cellular NADPH levels (32, 33). *Hmgb1* has been shown to be released by neuronal cells following EtOH stimulation and can promote the further release of proinflammatory factors (34). Combined with the fact that *Panx1*, a component of gap junctions, is enriched in S-type astrocytes, *Hmgb1* as a releasable factor could also play a role in S-type regulation of overall responsiveness through intercellular communication. Mitochondrial function: forty-five genes differentially expressed between S- and T-types are responsible for mitochondrial function (Fig. 4*A*: mitochondria, Table S5), including enzymes that metabolize EtOH (*Aldh2*), enzymes responsible for oxidative phosphorylation or its regulation (cristae: *Adck1*, uncoupling: *Slc25a47,* complex I: *Ndufb4, Ndufs8, Nubpl;* complex II: *Sdhc;* complex III: *Bcs1l*; complex IV: *Cox6c, Cox7b, Coa4, Taco1;* ATP synthase: *Atp5f1*), mitochondrial proteostasis (transport: *Rab3d, Timm10b, Timm17a, Timm44*; translation: *Mrpl1, Mrpl52, Mrpl13, Mrpl20, Rpl35a, Wars2, Secisbp2, Trmt61b*; processing: *Immp1l, Hsp90ab1*), and lipid metabolism (*Abca8b*, *Lipt2*, *Acad8, Tango2, Ptpmt1, Acp6, Cerk, Mboat7*) (Fig. 4*B*: mitochondrial). Since mitochondrial metabolism is intimately linked to mitochondrial calcium dynamics, this may contribute to differential calcium responses observed between S- and T-types. Regulation of mitochondrial calcium stores and corresponding intracellular calcium levels complexly interact under homeostasis and pathology. However, the examination of EtOH-induced genes one hour after exposure did not identify apoptotic genes activated by the mitochondria (Table S2), further supporting the fact that subtype-specific EtOH-induced response properties fall within the physiological responsiveness. Intracellular calcium: Forty-seven genes controlling calcium release from internal stores and channels at the extracellular membrane are differentially expressed between the S- and T-type astrocytes (Fig. 4*A*: Calcium, Table S5), some of which overlap with mitochondrial functions (Fig. 4*B*: Calcium). For example, *Vdac1*, which can release calcium from mitochondria under specific scenarios (35, 36, 37), is increased in expression in T-types relative to S-types, whereas genes that regulate calcium release from other internal stores are enriched in S-types (*Gpr30*, *Orai2*, *Plcb1*, *Ptgfr*, *Stim2*). In addition, two subunits that regulate L-type voltage-gated calcium channels, known to be expressed and functional in astrocytes (38, 39, 40, 41), are more highly expressed in S-types relative to T-types (*Cacnb3*, *Cacnb2*). Though not voltage dependent, *Slc8a1*, also known as *NCX1*, is also enriched in S-types, and under certain conditions, the activity of this transporter can result in net calcium influx (42, 43). Differential expression of these calcium flux regulators and mitochondrial proteins at baseline indicates the implementation of different gene expression programs with regards to mitochondrial and calcium regulation revealed by baseline EtOH-induced calcium responses.

Besides gene expression differences related to calcium signals, potential epigenetic mechanism difference between S- and T-type astrocytes were identified. This category includes genes whose function modifies gene expression, including transcription factors and associated regulators of transcription and chromatin remodeling factors (Fig. 4*B*, lower panel). Differential expression was found between S- and T-types for a number of genes that use SAM as a cofactor for methylation of targets other than DNA (RNAs: *Rngtt*, *Bcdin3d*; proteins: *Mettl18*, *Prmt8*; metabolites: *Gamt*, *Hnmt*) (Fig. 4*A*: epigenetic, Table S5), pointing at baseline differences in 1-carbon metabolism that could impact chromatin modifications and structure and subsequently gene expression (44, 45, 46, 47, 48). As well as a number of transcription factors whose activity can directly regulate the transcription of other genes (*Hivep1*, *E4f1*, *Zeb1*, *Sox9*, *Zfp37*, *Nfyb*, *Atf5*, and *Mafb*) (Fig. 4*B*: transcription regulators), many genes involved in chromatin structure and organization were differentially expressed between S- and T-type astrocytes (Fig. 4*B*: Chromatin remodelers). These genes include factors that regulate chromatin structure through direct histone H2A, H3, or H4 acetylation (*Kansl2*, *Brd8*, *Brpf3*, *Ncoa3*) or recruitment of acetylating enzymes (*Npat*) as well as deacetylation (direct: *Phf21a*, *via* enzyme recruitment: *Lrif1, Mtf2, Phf19*). Other DEGs directly methylate lysine residues within histones H3 or H4 (*Suv420h2*, *Suv39h2*, *Whsc1*, *Yaf2*, *Ehmt1*) or recruit enzymes that do so (*Wiz*, *Mllt10*, *Paf1*). Additional genes bind to methylated histones to activate (*Hmgn3*) or repress (*L3mbtl2*, *L3mbtl3*, *Mbd4*, *Cbx5*, *Asxl2*) transcription. Furthermore, a subset of these genes controls the ubiquitination status of histones (*Paf1*, *Pcgf6*, *Mbd5*).

### High read depth sequencing enables rich characterization of calcium response associated gene signatures not previously identified in the literature

DEGs were identified between S- and T-type astrocytes using high depth sequencing at least 30 times higher than existing high throughput single-cell methods in which prior individual phenotypic status is unknown (49, 50, 51, 52, 53, 54, 55, 56). Moreover, our method results in over 10,000 detected genes, ∼5 times greater than in existing single-cell RNA-seq datasets (49, 53, 56, 57) (Fig. S3*A*). This gene detection sensitivity is crucial in detection of genes with low expression and facilitating their comparison between different conditions, especially when there is a subtle difference in expression levels. Indeed, by examining the proportion of cells that have nonzero expression of the T- *versus* S-type genes, none of the three publicly available single-cell RNA-seq astrocyte datasets exceed 20% detection, while the process described here achieved detection of over 80%, which is almost comparable to the bulk RNA-seq counterpart by Erickson *et al.* (Fig. S3*B*). Furthermore, gradual downsampling our data from one million to 10,000 total reads in the S, T, M, and NR samples resulted in a rapid decrease in gene detection sensitivity, which approaches zero when the library size is reduced to as low as that in Zeisel *et al.* (10,000), a point at which the specificity is almost halved (from 89% to 50%, Fig. S3*C*). Although supervised clustering based on calcium signal type still shows good subtype discriminability (Fig. S3*D*), the ability to identify DEGs is markedly reduced (49).

**Figure 5:**
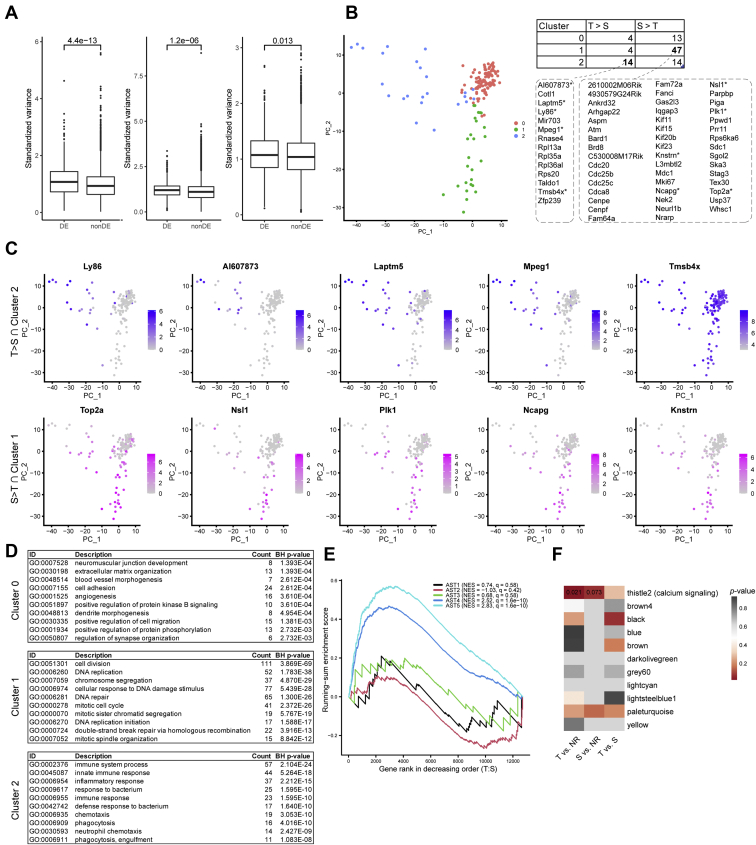
**Validation of the T- and S-types using external mouse and human astrocyte transcriptome datasets.***A*, boxplots comparing the standardized variance of the T- *versus* S-type differential (DE) and nondifferential (nonDE) genes in three public mouse astrocyte single-cell RNA-seq datasets: Spaethling *et al.* (*left*), Batiuk *et al.* (*middle*), Vanlandewijck *et al.* (*right*). *B*, left: PCA clustering of the mouse astrocytes from Spaethling *et al.* into three clusters. *Right*: overlap of the T *versus* S genes from this study and the cluster-specific genes from Spaethling *et al.* Genes shared between T- > S-type and Cluster 2, and between T- < S-type and Cluster 1 are listed at the *bottom*, respectively. *C*, expression of the top five cluster-specific genes (with *asterisks* in B) in Spaethling *et al. D*, *top* 10 GO enriched functions in Cluster 0, 1, and 2 from Spaethling *et al. E*, GSEA running-sum enrichment scores of the T *versus* S differential expression against the five astrocyte clusters (AST1-5) defined by Batiuk *et al. F*, heatmap showing the overlap between the subtype differentiating genes and the human alcohol dependency modules (size > 20) defined by Kapoor *et al.* Texts show the *p*-values for two marginally significant overlap. GO, gene ontology; GSEA, gene set enrichment analysis; PCA, principal component analysis.

Due to the technical differences from public datasets such as RNA amplification methods and sequencing read depth, direct validation of identified DEGs across different calcium signal types in this study by an existing dataset is not ideal. Instead, we tested the variances of gene expression in identified DEGs and stationary genes (non-DEGs) from S- and T-type astrocytes in public astrocyte transcriptome datasets (53, 56, 58) (Fig. 5*A*). The significant difference in variance of those two gene populations were observed in other single astrocyte transcriptome studies using *in vitro-* as well as *in vivo*-derived astrocytes samples (Fig. 5*A*). Using the cultured astrocyte single-cell transcriptome dataset (58), we identified three different classes of astrocyte subgroups. The clusters represent features of cell cycle–related function enrichment (cluster 1) and immune-related function enrichment (cluster 2) (Fig. 5, *B* and *D*). The pronounced genes overlapping in different clusters with response type preferences was observed with 47 genes in cluster 1 shared with S-type and 14 genes in cluster 2 with T-type astrocytes (Fig. 5*B*). For example, the increased immune-related transcripts (*Ly86* and *Mpeg1*), dominantly expressed in T-type astrocytes, were primarily expressed in cluster 2 with lower expression in other clusters. The opposite trend was observed with S-type signature genes in cluster 1 (Fig. 5*C*). This unsupervised clustering method was able to cluster the *in vivo* mouse brain vascular single-cell dataset (56), where we detected two major classes (cluster 0 and 1) of astrocytes (Fig. S4*A*). Despite the dissimilarity with our data in terms of the differential genes identified, functionally, we observed the GO term, *the mitotic spindle pole*, in cluster 0 (Fig. S4*B*). Based on type-specific DEGs, we compared our *in vitro* results to the *in vivo* study by Batiuk *et al*., which established five distinct astrocyte clusters based on transcriptomes from acutely isolated cells in the adult mouse brain (53). The T-type–specific genes were exclusively enriched in the astrocyte subgroup AST4 and AST5 (Fig. 5*E*), which are reported to share the features of the neural stem or progenitor cells and the intermediate progenitors, respectively (53).

To assess the relevance of translation of results from mouse to the human, DEGs in this study was compared to the human alcoholism transcriptome dataset. Eleven human alcohol dependency coexpression modules with more than 20 genes were extracted from the study by Kapoor *et al*. A hypergeometric test quantified their overlap with our astrocyte type-specific DEGs. As shown in Figure 5*F*, the T-type–specific DEGs (against the NR baseline) show moderately significant (*p* = 0.021) overlap with the thistle2 module, which is mapped to networks correlated to calcium signaling functions. In comparison, the S-type–specific DEGs (against NR) show marginally significant (*p* = 0.073) enrichment (59).

In summary, the higher gene expression variances in type-specific DEGs identified here in other astrocyte transcriptome studies supports the existence of EtOH-sensitive astrocyte subgroups. The approach of combining physiological assessment as a selection factor for gene expression analysis and high read depth single-cell RNA-seq for coverage of baseline gene expression in this study are critical elements to achieve the required sensitivity of detection of these gene profiles in astrocytes. The association of those gene profiles with calcium signals is essential to define functional astrocyte subgroups, which is not yet established, and is an essential initial step in the long path to understanding the role of astrocytes in alcohol abuse–related disorders.

## Discussion

Due to the intrinsic chemical properties of EtOH as a small and polar molecule, even with clear pathological consequences of overuse, numerous biological processes can be altered beyond conventional drug-receptor interactions. EtOH broadly influences cellular activity and subsequent higher brain function in the nervous system, but its effect on astrocytes and the role of astrocytes under EtOH exposure is not well characterized. In this study, we investigate the heterogeneity of the astrocyte phenotype using a combination of physiology and genomics tools to understand the EtOH responsiveness in astrocyte subgroups. Using a controllable *in vitro* primary cell culture system, subsets of astrocytes that exhibited a distinct phenotype were identified based on calcium responses to acute EtOH exposure (S-, T-, M-, and NR types). These response types were observed in response to a range of EtOH doses and persisted with repeated EtOH exposure, suggesting they represent a stable phenotypic trait. These findings were further supported by classifying distinct gene signatures associated with the response types with the least inter-group variability: S- and T-types. It is important to note that the high per gene read coverage achieved with deep sequencing of samples played a vital role in identifying these understated gene signatures. Many of the high-throughput approaches for single-cell RNA-seq, including droplet-based cell separation, result in the multiplexing of hundreds to thousands of single cells onto a single sequencing lane, typically ensuing in the capture of many samples at low read coverage and thus low gene detection (60, 61, 62, 63). While high-throughput approaches are amenable to identifying highly enriched genes that distinguish between major cell types among massive quantities of cells, the loss of the capability to identify low abundance genes limits interpretability of functional markers, especially among cells with nondominant phenotypes, that is, rare cell types. The complementary approach in this study uses deep sequencing and ion-imaging, allowing the detection of unknown EtOH-sensitive astrocyte subgroups and confirming subtle gene expression signatures by the discovery of a significant number of genes across subgroups.

The complex expression patterns of many genes are the result of nucleosome modifications that result in either direct or indirect effects on gene expression, and thus chromatin modifications and resulting structure are intimately linked to and indicative of changes in gene expression (64). These long-term chromatin modifications are generally discussed in the context of stem or progenitor cells as they advance through the process of terminal differentiation. However, astrocytes of adult mammals are still able to divide *in vitro* and *in vivo* (65, 66, 67, 68)*,* indicating these differential chromatin modifications may be passed on to daughter cells and potentially alter brain circuit functions (69, 70, 71, 72, 73) over the long term. Apparently, a human post mortem brain tissue study shows alcoholism reduces the astrocyte population (74). In correlation with epigenetic DEGs in this study, it could suggest that the involvement of epigenetic modification in astrocytes by EtOH exposure might play potential roles in astrocyte fate. Interestingly, the shared functional enrichment of T-type DEGs in this study with *in vivo* astrocyte subgroups (AST4: neural stem or progenitor cells and AST5: intermediate progenitors) suggest potential type-specific alterations of an intrinsic molecular program (53). In addition, the difference in baseline expression of epigenetic genes between S- and T-type astrocyte also suggest a hypothetical subgroup-specific mechanism for maintaining heightened EtOH susceptibility even in the absence of EtOH usage.

Despite the vital role of calcium in brain function, the differential EtOH-induced astrocyte calcium responses identified here in the brain are unclear. DEGs discriminating between S- and T-types are enriched across mitochondrial functions, calcium homeostasis, and inflammation pathways. All of these pathways are closely related to calcium regulation in cells. Thus, the EtOH-induced calcium signal type might not be generated by conventional activation of specific pathways but due to the complex interactions of the cellular processes. Any modifications of the activation or distortion of those genes can potentially modify calcium signals in astrocytes. The strongest calcium signals present in M-type and the expression of downregulated genes in M-type astrocytes were similar to those in NR astrocytes. However, upregulated genes of M-type completely intersected with all EtOH other responsive astrocytes, suggesting that intersected genes across calcium responsive astrocytes share molecular factors for calcium signal generation. Potentially, the downregulated genes shared between M-type and NR specifically act to lower the threshold and eventually enforce the strongest calcium response to EtOH. In fact, in the coculture configuration in which there is a developmental and constant interaction between neurons and astrocytes, we found that repeated EtOH stimulation evokes reciprocal communication between neuron and astrocytes and enhances their calcium signal intensity through extracellular chemicals (24). With the astrocyte response type perspective in this study, the enhanced astrocyte calcium signal is the most indicative of M-type characteristics. Whether those calcium signal enhanced astrocytes show the same gene signatures as M-type or not, a simultaneous increase of calcium signal enhancement in both neuron and astrocyte subgroups indicate their concerted participation in EtOH response. Thus, EtOH-induced heterogenous calcium signals in astrocytes suggest that strategic distribution of types could actively and differentially influence the brain cell network through regulation of reciprocal intercellular signal strength upon stimulus-induced activity of calcium-dependent astrocyte functions.

The findings in this study have significant implications *in vivo* since these response-specific DEGs identified *in vitro* are associated with gene expression signatures in acutely isolated astrocytes from adult mouse brains, and those astrocytes also display distinct calcium signal properties (53). The correlation of identified DEGs and gene functions in the present study to the human alcoholic brain gene set implicates the potential translational investigation on the significance of calcium signal-related core gene sets in human alcohol use disorder and fetal alcohol syndrome (59). T-type astrocytes are the most distinctive subgroup from other astrocytes based on common calcium signal-related features of gene expression in both *in vitro* and *in vivo* as well as in human samples. Recently, it has been identified that some astrocytes nonresponsive to EtOH at lower doses are responsive at higher doses, while a certain percentage of astrocytes remain persistently nonresponsive at all EtOH doses tested (24). In addition to the characterization of the response-type–specific subtypes *in vivo*, it will be informative to determine the distinction in NR astrocytes that are persistently nonresponsive at all EtOH doses (*i.e.*, those defined as nonresponsive at the 100 mM dose) compared to NR astrocytes that are recruited to respond to EtOH at higher doses. (*i.e.*, those defined as nonresponsive at the lower doses but responsive at the 100 mM dose). This process of recruitment of nonresponsive to responsive cells with higher EtOH doses further highlights the potential role of astrocyte plasticity, which could modify the overall EtOH responsiveness in the brain. Therefore, investigating the differential gene expression of those two groups could reveal gene markers critical for EtOH susceptibility or resiliency in astrocytes. Brain region differences in S-, T-, M-, or NR type distribution *in vivo* could result in regionally distinct responses to EtOH exposure. Thus, the rich information of functionally linked DEGs and their association with calcium signal characteristics will provide the basis for future investigations of functional differences in different astrocyte subgroups. Understanding the role of each response type in EtOH-induced pathologies will be enriched by future studies that identify the role of EtOH-responsive genes in each response type associated with different susceptibility or resilience to the effects of EtOH. Manipulation of these factors *in vivo* will inform the ability to shape EtOH responses toward the resilient states (75). Our results also suggest that a cell subtype nomenclature, in which gene signatures are paired with phenotype parameters, may specifically define the unknown functional feature of astrocytes and other cells (76). Future studies will be important in dissecting these potential effects in a system closer to the *in vivo* state or *in vivo* in the context of modulation of the identified gene targets by characterizing the functional dependence or relationship between gene expression changes, physiological responsiveness, and resiliency of the EtOH impact on altering cellular functions and systemic malfunctions.

## Experimental procedures

### Animals

Timed pregnant C57BL/6 mice were purchased from Charles River. All procedures were approved by the University of Pennsylvania’s Institutional Animal Care Committee.

### Drugs and chemicals

Ethanol-200 proof (Decon Labs #2716) was obtained from Decon Labs through the Alcohol Service Center at the University of Pennsylvania. Other chemicals used in the recording solution for calcium imaging were purchased from Sigma.

### Primary mouse cortical astrocyte culture

Primary cultured astrocytes were prepared from cortex of single C57BL/6 mouse pups (P0-P1) as described (77). In brief, the cerebral cortex was dissected from the brain and adherent meninges were removed prior to mincing and dissociation with 2.5% trypsin (20 min, #15090-046, Gibco) by trituration. This single-cell suspension was then passed through a 40 μm cell strainer (#22363547, Fisher Scientific) to remove clumps. Cells were plated in 25 cm^2^ plastic flasks (#sial0639, Sigma) and cultured in Dulbecco’s modified Eagle’s medium (#10–013, Corning) supplemented with (in mM) 25 glucose, 4 L-glutamine, one sodium pyruvate, and 10% fetal bovine serum (#100-500, GemCell). Cultures were maintained at 37 °C in a humidified 5% CO_2_ incubator. The media was replaced every 2 to 3 days. Prior to replating on glass coverslips coated with 0.1 mg/ml poly L-lysine (#P9155, Sigma), cultures were shaken (250 rpm for 6 h) and treated for 12 h with cytosine arabinoside (10 μM) to remove potentially contaminating cell types such as microglia.

### Calcium imaging and sample collection

Coverslips containing plated astrocytes were loaded with 10 μg/ml Fluo-4 AM (#F14201, Thermo Fisher Scientific) mixed with 0.0001% pluronic acid (#P3000MP, Thermo Fisher Scientific) for 25 min at room temperature then de-esterified for 10 min with continual perfusion of external solution. Serum was removed 24 h prior to experiments. External solution (imaging saline, perfusion speed: 3 ml/min) contained 140 mM NaCl, 5.4 mM KCl, 1 mM MgCl_2_, 2 mM CaCl_2_, 16 mM glucose, 10 mM Hepes, adjusted to pH 7.3. The calcium indicator-loaded coverslip was then transferred to an open bath chamber (RC-27, Warner Instruments) for imaging and sample collection. To minimize cell-to-cell fluorescence loading variation and animal and culture batch variations, the calcium signal in individual astrocytes was normalized against calcium signals induced by 100 μM ATP at the end of each experiment. For single-cell isolation, targeted single cells were identified based on real-time imaging results and collected with a pipet tip pulled from a glass microcapillary (#3450099, Thermo Fisher Scientific) controlled by a micromanipulator (Eppendorf, Transferman NK2). Immediately after isolating samples by gentle aspiration, samples were snap frozen on an ethanol-dry ice mixture then briefly stored at −20 ^°^C prior to mRNA amplification (78).

### Calcium imaging and data analysis

All *in vitro* imaging experiments were performed using a Zeiss 710 meta upright laser-scanning microscope (W20x plan-apochromatic lens, 1.0NA). Briefly, settings for fluo-4 imaging were 488 nm laser excitation and 505 to 570 nm emission collection through a 488 nm long-pass dichroic mirror. A sub-stage detector was used to simultaneously collect reflected images for cell morphology assessments and micro pipet positioning. A standard imaging field of 1680 μm^2^ with 512 pixel^2^ resolution, 3.2 μs pixel dwell time, and 320 μm pinhole size was used. For faster imaging (1 Hz), bidirectional scanning mode was employed. All imaging experiments were performed at room temperature and continuously perfused with fresh saline. Based on these settings, one field of view contains approximately 25 astrocytes. In order to maintain the proper ethanol concentration in the cellular saline perfusate, fresh anhydrous 200 proof ethanol was used to prepare the desired concentration just before the start of an experiment. Zeiss Zen program was used to analyze calcium dynamics online for cell selection. Acquired time-lapse raw images (16 bit) were post processed using MetaMorph Offline version 7.8 (Molecular Devices) to extract intensity values and calculate intensity changes for confirmation of cell types. Briefly, a cell-free area of the coverslip was chosen for the background and subtracted from the entire image stack. Regions of interest in the cytosol were selected based on the mean pixel values across the time-collapsed image stack. For quantitative calcium signaling analysis, the temporal dynamics in fluorescence changes were expressed as background-subtracted values ΔF/F_o_ = ((F-F_o_)/F_o_), where F_0_ represents the fluorescence level of the cells before stimulation, and ΔF represents the change in fluorescence occurring following stimulation of the cell. The calculated ΔF in every single astrocyte was then normalized against individual maximal calcium responses induced by application of 100 μM ATP at the end of each experiment.

### RNA amplification, library construction, and sequencing

Single cells were collected in 5x first strand buffer (ThermoFisher, supplied with #1808004) and snap frozen prior to storage. Single cells were then thawed and amplified using modifications to the antisense RNA (aRNA) amplification procedure (78). Alterations included using Beckman Coulter Ampure XP beads (#A63881) for all cleanups after second strand reaction or the Beckman Coulter Agencourt RNAClean XP (#A63987) following the *in vitro* transcription reaction. Cleaned double stranded cDNA or cleaned aRNA was eluted in small volumes to allow direct input into the next step without the need for an ethanol precipitation or speed vacuum concentration step. Amplification was restricted to two rounds to ameliorate the shortening effect of random primed-based approaches as well as the severity of 3′bias of polyA selection amplification approaches. Second round aRNA was sheared based on overall size distribution assessed using an Agilent Tapestation 2200, with the shearing time adjusted for longer average aRNA products, prior to input into the Illumina TruSeq mRNA stranded library construction procedure (#20020594, set A indexes #20020492 and set B indexes #20020493). A total of 50 ng of aRNA from a single sample was input into library construction. All other steps after shearing were followed according to the manufacturer’s specifications. Following completion of library preparation, samples were multiplexed at 12 samples per lane and sequenced on a NextSeq 500 using the 150 cycle high output v2 kit (#FC-404-2002), allowing for paired end 2 x 75 base pair reads.

### Read processing and computational analysis

Following sequencing, adapters, library barcode sequences, polyA tails, and low-quality bases (phred score < 20) were trimmed from all reads using a custom python script prior to alignment to the mouse genome (GENCODE GRCm38) using STAR (https://github.com/safisher/ngs/blob/master/ngs_STAR.sh). Feature counts of high quality uniquely aligned reads were quantified using VERSE (modified HTSeq with hierarchical assignment: exon, mito, intron, intergenic) (28). The raw count matrix and the metadata can be found in Table S1. Raw exonic read counts were normalized using DESeq (29) and genes expressed in less than five cells were filtered out. For the comparison of S-, M-, T-type, and NR astrocytes, the DESeq2 Wald test for differential expression was performed in R and statistical output was obtained for all contrasting datasets. Significant genes were retained where FDR < 0.05 and foldchange ≥ 2 or ≤ 0.5. To identify potential upregulation of apoptotic genes by the treatment scheme used, the Likelihood Ratio Test was employed in the DESeq2 R package to find genes whose expression was altered over time in a response-dependent manner. The reduced model that includes the response type and collection time point was tested against the full model that also includes the interaction between response and time. Significant genes were retained where FDR < 0.1. Volcano plots of significant genes which visually contrast the differential expression analysis of the DESeq2 Wald test were generated with the R package ggplot2 (79). Heatmaps were generated with the R package pheatmap (80). For the external single-cell RNA-seq datasets, R package Seurat 4 (81) was used for data import, normalization and scaling, dimension reduction (PCA, UMAP, t-SNE), and Louvain module detection. GO enrichment and GSEA were done with the R package clusterProfiler (82). UpSet diagrams were done with the R package UpSetR (83).

### Statistics

Data are presented as mean ± SD. For all experiments, data normality was first assessed using a Shapiro-Wilk normality test. For normally distributed data, differences between groups were evaluated by unpaired two-tailed t-tests or one-way ANOVA with Dunnett's multiple comparisons post-hoc tests. For data not following a normal distribution, Mann-Whitney tests (Two-tailed) were performed. Sigmaplot 12.0 and MS Excel were used for these analyses and to create the plots. Additional statistical details are provided in Table S6.

## Data availability

The data that support the findings of this study are available in Gene Expression Omnibus (GEO) repository at NCBI, reference number GSE199025.

## Supporting information

This article contains supporting information (49, 53, 56, 57).

## Conflict of interest

The authors declare no competing interests.

## Figures and Tables

**Supporting infomation_Figure 1:**
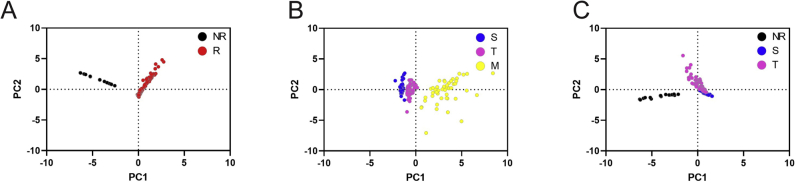


**Supporting infomation_Figure 2:**
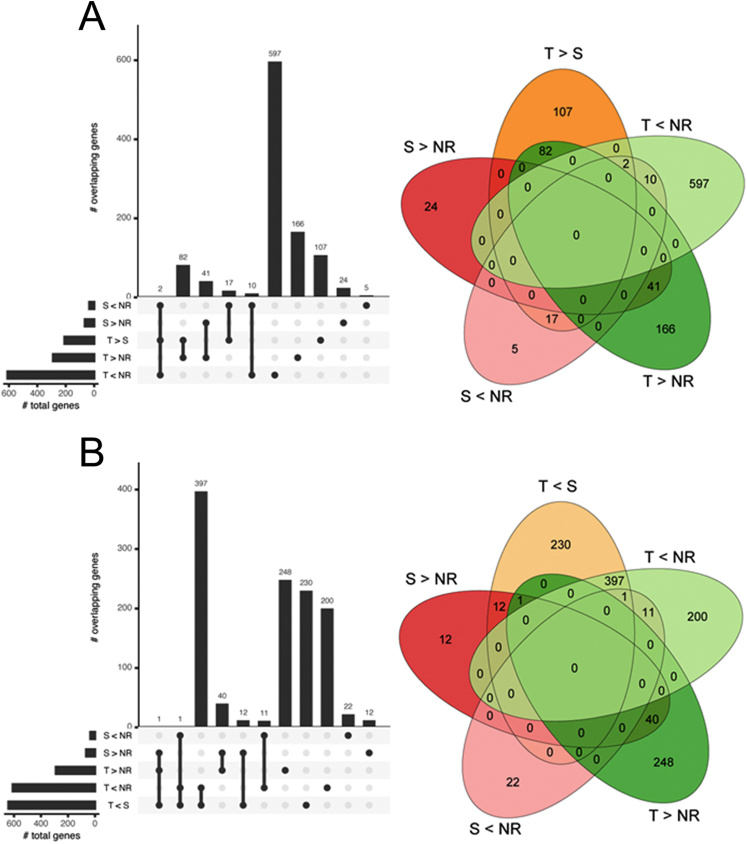


**Supporting infomation_Figure 3:**
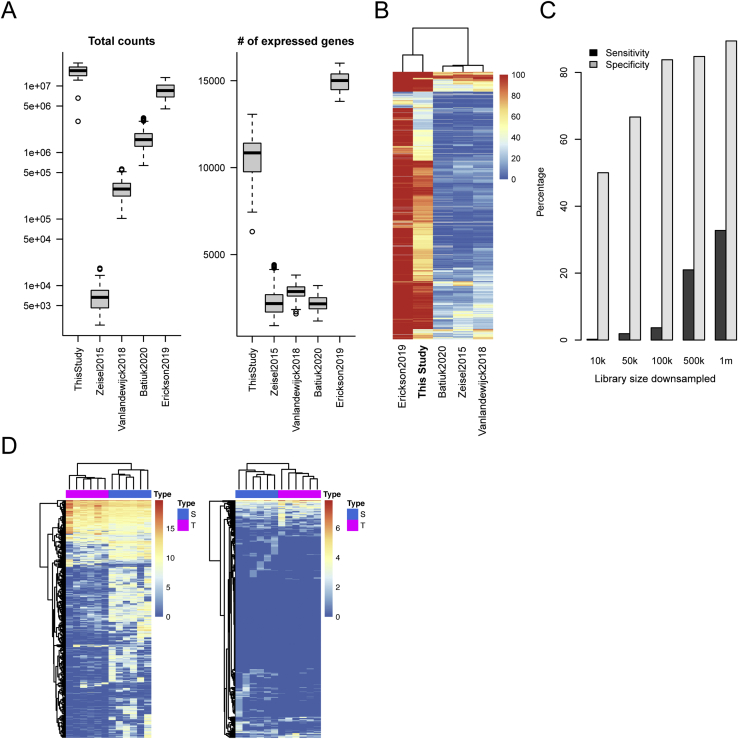


**Supporting infomation_Figure 4:**
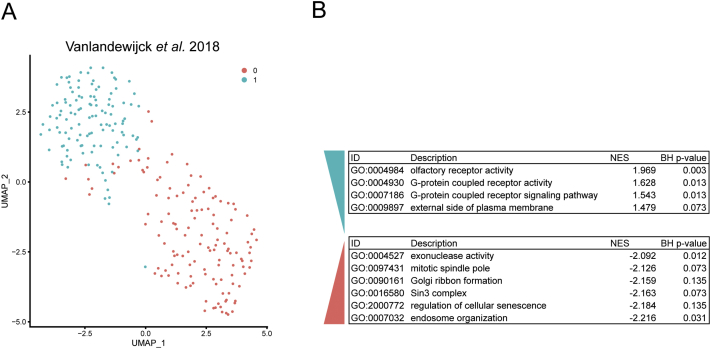


## Supporting information

supporting information text

Supporting infomation_Table S1

Supporting infomation_Table S2

Supporting infomation_Table S3

Supporting infomation_Table S4

Supporting infomation_Table S5

Supporting infomation_Table S6
