# Supplementary material for: Astrocyte ethanol exposure reveals persistent and defined calcium response subtypes and associated gene signatures
Source: J Biol Chem. 2022 Jun 16;298(8):102147. doi: 10.1016/j.jbc.2022.102147 (PMC9293641; doi:10.1016/j.jbc.2022.102147)
Supplement: supporting information text [file mmc1.docx]

**Figure S1. Classification of EtOH induced astrocyte calcium responses and subtype identification.**

In order to define a calcium response as EtOH-induced, the % changes in normalized ΔF over baseline (before EtOH application and saline application) from all the samples were used to identify the EtOH-induced calcium signal without bias. Two clusters were identified by multiple regression analysis as high and low variable groups. Using average and standard deviation (SD) of the low variable group, a cut-off for non-responsive was defined as less than 20% change (over than AVE + (2 x SD) of low variable group). Based on calcium waveform characteristics such as slope, amplitude, and frequency, classification parameters were set and tested using principal component analysis (PCA) for validation. **A**, Clustering between EtOH-responsive (R) versus Non-responsive (NR). **B**, S-,T- and M-type clustering. **C**, Non-responsive (NR) versus S-and T-type astrocytes. All detailed information for PCA is in Table S6.

**Figure S2.** **Examination of the regulatory direction of the T vs. S differential genes with respect to the T vs. NR or S vs. NR comparisons.**

**A**, UpSet plot and Venn diagram showing the overlap between T- > S-type genes and the T- or S-type differential (up- or down-regulated) genes in contrast to the NR baseline. **B**, UpSet plot and Venn diagram showing the overlap between T- < S-type genes and the T- or S-type differential (up- or down-regulated) genes in contrast to the NR baseline.

**Figure S3. Assessment of the sequencing depth-statistical power relationship in S- and T-type gene signatures using mouse astrocyte transcriptomes from Erickson *et al.,* (59), Batiuk *et al.,* (55), Zeisel *et al.*, (51)*,* and Vanlandewijck *et al.*, (58).**

**A**, Boxplots showing the total read counts (left) or the number of expressed genes (right) in this study and in the four public mouse astrocyte transcriptome datasets. **B**, Heatmap showing the percentage of cells expressing the T vs. S differential genes in this study and the four public datasets in comparison except Erickson *et al.* For Erickson *et al.,* in which the percentage of samples is displayed. Columns are clustered by the Euclidean distance between each study. **C**, Barplots showing the sensitivity (black) and the specificity (grey) when the sequencing depth of each cell in this study is downsampled to one million (1m), 500,000 (500k), 100,000 (100k), 50,000 (50k) and 10,000 (10k) exonic reads. **D**, Heatmaps showing the T vs. S differential genes’ normalized expression levels in T- and S-types given the original sequencing depth (left) and the downsampled sequencing depth (right). Cells are hierarchically clustered by the Euclidean distance.

**Figure S4. Astrocyte subtype detection and GO functional enrichment in Vanlandewijck *et al.*’s (58) mouse astrocyte transcriptome data.**

**A**, UMAP visualization of the mouse astrocytes from Vanlandewijck *et al.* Colors represent two clusters identified by Louvain module detection. **B**, GSEA significant enrichments in Cluster 0 (red) or Cluster 1 (green) specific genes.

**Table S1.** Raw count matrix for analysis.

**Table S2.** Canonical apoptotic gene expression analysis of single astrocytes.

**Table S3.** List of DEGs between calcium response types T, S and NR.

**Table S4.** Gene Ontology enrichment analysis of T- vs. S-type *unique* or *shared* differential genes and GSEA significant terms for T- vs. S-type differential genes.

**Table S5.** Gene Ontology enrichment analysis and GSEA significant terms for T- vs. S-type, T- vs. NR-type, S- vs. NR-type and M- vs. NR-type differential genes**.**

**Table S6.** Statistical analysis results for Figure 1 and S1.
