# Supplementary material for: Astrocyte ethanol exposure reveals persistent and defined calcium response subtypes and associated gene signatures
Source: J Biol Chem. 2022 Jun 16;298(8):102147. doi: 10.1016/j.jbc.2022.102147 (PMC9293641; doi:10.1016/j.jbc.2022.102147)
Supplement: Supporting infomation_Table S6 [file mmc7.docx]

**Table S6. Results from statistical analysis.**

| Figure. No. | Results from statistical analysis |  |
| --- | --- | --- |
| Figure 1A | ***Astrocyte***  No response:  (Fmax (Normalized, %) = 2.5175 ± 1.3324,  **Δ** F (%) = 105.270 ± 11.9967, n = 77);  Response:  (Fmax (Normalized, %) = 17.0179 ± 16.6566,  **Δ** F (%) = 348.3120 ± 29.0260, n = 144);  Fmax (Normalized, %)  Unpaired two-tailed t-test, Power of performed test with alpha = 0.050: 1.000:  t = 7.721, *P* < 0.001:  **Δ** F (%)  Unpaired two-tailed t-test, Power of performed test with alpha = 0.050: 1.000:  t = 5.622, *P* < 0.001. |  |
| Figure 1B | ***Astrocyte***  Type S, EtOH 100 mM:  (Fmax (Normalized, %) = 5.1590 ± 1.7413,  Slope = 0.0122 ± 0.0275, AUC (Normalized, %) = 18.7161 ± 1.3011, n = 94);  Type T, EtOH 100 mM:  (Fmax (Normalized, %) = 10.8570 ± 4.0020,  Slope = 0.5280 ±0.3935, AUC (Normalized, %) = 24.25891 ± 1.3438, n = 64);  Type M, EtOH 100 mM:  (Fmax (Normalized, %) = 41.9006 ± 13.9099,  Slope = 3.3268 ± 2.0499, AUC (Normalized, %) = 32.24847 ± 2.9587, n = 47).  ***S vs T PCA (Principal Component Analysis)***  PC1  Eigenvalue = 3.651  Proportion of variance = 45.64%  PC2  Eigenvalue = 1.894  Proportion of variance = 23.67%  Component selection method: Parallel analysis  Random seed = 525692468  Rows analyzed (# cases) =126 |  |
| Figure 1C | ***Astrocyte Responsive cells (%)***  Saline (1.2500 ± 2.5000, Coverslips =4, n = 62),  EtOH 5 mM (13.4300 ± 11.2470, Coverslips = 5, n = 88),  EtOH 10 mM (12.7823 ± 11.2254, Coverslips = 18, n = 291),  EtOH 50 mM (20.0233 ± 10.8082, Coverslips = 11, n = 247),  EtOH 100 mM (78.3083 ± 5.5997, Coverslips = 8, n = 125),  One-way ANOVA with Dunnett's multiple comparisons test, F (4,42) = 76.251, *P* < 0.001:  EtOH 100 mM vs. Saline, ^###^ *P* < 0.001;  EtOH 100 mM vs. EtOH 5 mM, *** *P* < 0.001;  EtOH 100 mM vs. EtOH 10 mM, *** *P* < 0.001;  EtOH 100m M vs. EtOH 50 mM, *** *P* < 0.001*.*  One-way ANOVA with Holm-sidak comparisons test, F (3,32) =1.701, *P* = 0.1.999:  EtOH 5 mM vs. EtOH 10 mM, *P* = 0.909;  EtOH 5 mM vs. EtOH 50 mM, *P* = 0.446;  EtOH 10m M vs. EtOH 50 mM, *P* = 0.224*.* |  |
| Figure 1C | ***Astrocyte Type composition***  EtOH 5 mM:  Type S = 6.3830 ± 1.4072,  Type T = 4.2636 ± 1.2868,  Type M = 3.1915 ± 1.4502,  EtOH 10 mM:  Type S = 9.0741 ± 2.1072,  Type T = 2.3742 ± 0.9868,  Type M = 1.3339 ± 0.7504,  EtOH 50 mM:  Type S = 9.1946 ± 1.1561,  Type T = 4.6354 ± 1.3519,  Type M = 6.1933 ± 2.9358,  EtOH 100 mM:  Type S = 32.6374 ± 1.1561,  Type T = 27.5391 ± 3.0498,  Type M = 18.1319 ± 4.4123. |  |
| Figure 1D | ***Astrocyte Fmax (Normalized, %)***  Type NR:  EtOH 5 mM, Type NR = 2.6116 ± 1.6016,  EtOH 10 mM, Type NR = 2.5904 ± 1.4755,  EtOH 50 mM, Type NR = 2.5933 ± 1.3861,  EtOH 100 mM, Type NR = 2.6074 ± 1.5806,  One-way ANOVA with Dunnett's multiple comparisons test, F (3,359) = 0.0237,  EtOH 100 mM vs. EtOH 50 mM, ns *P* = 0.952;  EtOH 100 mM vs. EtOH 10 mM, ns *P* = 0.894;  EtOH 100 mM vs. EtOH 5 mM, ns *P* = 0.829.  Type S:  EtOH 5 mM, Type S = 3.7732 ± 0.4839,  EtOH 10 mM, Type S = 3.8215 ± 0.6033,  EtOH 50 mM, Type S = 4.2636 ± 0.5430,  EtOH 100 mM, Type S = 4.4813 ± 1.8096,  One-way ANOVA with Dunnett's multiple comparisons test, F (3,140) = 3.044,  EtOH 100 mM vs. EtOH 50 mM, ns *P* = 0.269;  EtOH 100 mM vs. EtOH 10 mM, ns *P* = 0.590;  EtOH 100 mM vs. EtOH 5 mM, ns *P* = 0.999.  Type T:  EtOH 5 mM, Type T = 7.3370 ± 3.4047,  EtOH 10 mM, Type T = 8.7330 ± 2.3360,  EtOH 50 mM, Type T = 8.5940 ± 3.3906,  EtOH 100 mM, Type T = 8.7330 ± 2.3360,  One-way ANOVA with Dunnett's multiple comparisons test, F (3,61) = 0.638,  EtOH 100 mM vs. EtOH 50 mM, ns *P* = 0.504;  EtOH 100 mM vs. EtOH 10 mM, ns *P* = 0.680.  EtOH 100 mM vs. EtOH 5 mM, ns *P* = 1.000.  Type M:  EtOH 5 mM, Type M = 27.5360 ± 3.0579,  EtOH 10 mM, Type M = 26.8010 ± 5.1723,  EtOH 50 mM, Type M = 31.1763 ± 15.3587,  EtOH 100 mM, Type M = 41.9006 ± 13.9099,  One-way ANOVA with Dunnett's multiple comparisons test, F (3,62) = 3.638, P = 0.018  EtOH 100 mM vs. EtOH 50 mM, ns *P* = 0.116;  EtOH 100 mM vs. EtOH 10 mM, ns *P* = 0.324;  EtOH 100 mM vs. EtOH 5 mM, ns *P* = 0.048. |  |
| Figure 1E | ***Astrocyte Pattern similarity (%, 1^st^ versus 2^nd^)***  Type S – Type S (91.8427 ± 5.9195, n = 193),  Type T– Type T (98.5507 ± 2.2452, n = 193),  Type M– Type M (94.6979 ± 6.2573, n = 193),  Total (95.0304 ± 6.2553, n = 193). |  |
| sFigure 1 | ***NR vs R PCA (Principal Component Analysis)***  PC1  Eigenvalue = 2.044  Proportion of variance = 51.09%  PC2  Eigenvalue = 1.744  Proportion of variance = 43.61%  Component selection method: Parallel analysis  Random seed = 524645984  Rows analyzed (# cases) =204  ***NR vs S vs T PCA (Principal Component Analysis)***  PC1  Eigenvalue = 2.475  Proportion of variance = 61.88%  PC2  Eigenvalue = 1.302  Proportion of variance = 32.56%  Component selection method: Parallel analysis  Random seed = 525445406  Rows analyzed (# cases) =157  ***S vs T vs M PCA (Principal Component Analysis)***  PC1  Eigenvalue = 4.762  Proportion of variance = 52.92%  PC2  Eigenvalue = 1.572  Proportion of variance = 17.47%  Component selection method: Parallel analysis  Random seed = 525026000  Rows analyzed (# cases) =172 |  |
|  | | |
